# 4,5-Seco-Guaiane and a Nine-Membered Sesquiterpene Lactone from *Holostylis reniformis*

**DOI:** 10.3390/molecules171214046

**Published:** 2012-11-27

**Authors:** Marcos D. P. Pereira, Tito da Silva, Lucia M. X. Lopes, Antoniana U. Krettli, Lucas S. Madureira, Julio Zukerman-Schpector

**Affiliations:** 1 Institute of Chemistry, São Paulo State University, UNESP, C.P. 355, 14801-970, Araraquara, SP, Brazil; E-Mails: mpelicon@bol.com.br (M.D.P.P.); titodasilv@hotmail.com (T.S.); 2 Laboratory of Malaria, Institute René Rachou/FIOCRUZ, Av. Augusto de Lima 1715, 30190-002, Belo Horizonte, MG, Brazil; E-Mail: akrettli@cpqrr.fiocruz.br; 3 Department of Chemistry, Federal University of São Carlos, C.P. 676, 13565-905, São Carlos, SP, Brazil; E-Mails: lucassousamadureira@gmail.com (L.S.M.); julio@power.ufscar.br (J.Z.-S.)

**Keywords:** *Holostylis reniformis*, Aristolochiaceae, sesquiterpene, 4,5-seco-guaiane, megastigmane

## Abstract

Root extracts of *Holostylis reniformis* (Aristolochiaceae) yielded three new natural sesquiterpenes, a sesquiterpene with an unusual carbon skeleton, 4,5-seco-guaiane (7-*epi*-11-hydroxychabrolidione A, **1**), a nine-membered lactone with new carbon skeleton (holostylactone, **2**), and a new megastigmane [(6*S*,7*E*)-6,9-dihydroxy-10-(2'-hydroxy-ethoxy)-4,7-megastigmadien-3-one, **3**], together with bulnesol and sitosterol-3-*O*-β-D-glucopyranoside. The structures of these compounds were determined by spectroscopic analyses and B3LYP/STO-3G** theoretical studies.

## 1. Introduction

*Holostylis reniformis *Duch. (Aristolochiaceae) has been used in traditional Brazilian medicine as an antirheumatic, stomachic, and depurative [1]. The anti-*Plasmodium falciparum* activities and toxicities of compounds and extracts obtained from this species have been demonstrated [2,3]. Since aryltetralone and furan lignans were the main chemical constituents found in the most active extracts, research has been concentrated on their chemical characterization [3,4,5], biosyntheses [6,7], and microbial transformations [8]. As part of our continuing studies on the chemical constituents of *H. reniformis*, in this paper the terpenoids obtained from the hexane and acetone extracts of its roots are reported.

## 2. Results and Discussion

**Figure 1:**
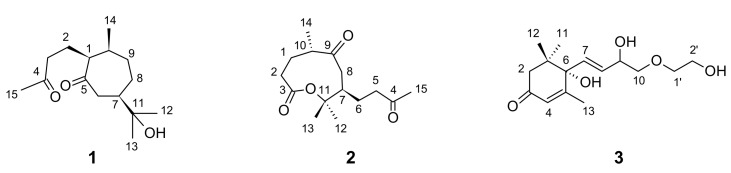
Chemical structures of compounds **1**–**3**.

The hexane extract of *H. reniformis* roots was subjected to column chromatography and yielded bulnesol, which was identified by comparison of its spectroscopic data with those reported in the literature [9,10], a 4,5-seco-guaiane **1** with an unusual carbon skeleton (Figure 1), and a nine-membered sesquiterpene lactone **2** with a new carbon skeleton (Figure 1). Together with sitosterol-3-*O*-β-D-glucopyranoside, which was identified by comparison of its spectroscopic data with those reported in the literature [11], a new sesquiterpene, megastigmane (**3**), was isolated from the acetone extract of roots (Figure 1). The structures of the new sesquiterpenes were determined by spectroscopic analyses and B3LYP/STO-3G** theoretical studies that were also performed.

**Figure 2:**
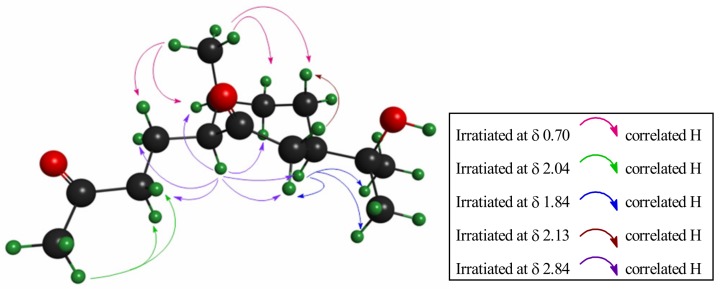
Selected nOe interactions for sesquiterpene **1**.

HRMS of compound **1** showed a *quasi*-molecular ion [M+Na]^+^ at *m/z* 277.1774 (C_15_H_26_O_3_+Na) and the MS-MS of these ions gave a base peak at *m/z* 237.18 [M−H_2_O+H]^+^. The ^13^C-NMR spectra of **1** showed signals for 15 carbons, including two carbonyl groups at δ 213.4 and 208.8, the latter of which corresponded to a methyl ketone (δ_H_ 2.04; δ_C_ 29.8), and a quaternary carbinolic carbon (δ 73.0) (Table 1). The presence of carbonyl and hydroxyl groups in the structure was also evidenced by absorption bands observed in the IR spectrum at 1707 and 3440 cm^−1^, respectively. Moreover, *g*HSQC, *g*HMQC, and DEPT 135° and 90° experiments evidenced the presence of an additional three methyl, five methylene, and three methine groups. The hydrogen chemical shifts of **1** were established with the aid of correlations observed by ^1^H–^1^H COSY, selective proton decoupling (HOMODEC), and *g*HMQC experiments. The *g*HMBC spectrum showed correlations between the carbonyl carbon signal at δ_C_ 208.8 (C-4) and the methyl hydrogens at δ_H_ 2.04 (3H-15) and the methylene hydrogens at δ_H_ 2.37 and 2.19 (2H-3), whereas, the carbonyl carbon signal at δ_C_ 213.4 (C-5) was correlated to H-6β (δ_H_ 2.13). Both C-7 (δ_C_ 46.4) and C-11 (δ_C_ 73.0) were shown to be correlated to the hydrogens of two methyl groups (δ_H_ 1.13 and 1.10). ^1^H–^1^H COSY experiment showed couplings between H-7 (δ_H_ 1.84) and H-6α (δ_H_ 2.52), H-6β (δ_H_ 2.13), H-8α (δ_H_ 1.75), and H-8β (δ_H_ 1.10), as well as between H-3a (δ_H_ 2.37) and H-3b, (δ_H_ 2.19), H-2a (δ_H_ 1.41), and H-2b (δ_H_ 1.13). The latter (H-2a,b) and H-8β were correlated to C-10 (δ_C_ 34.9), as well as 3H-14 (δ_H_ 0.70) were correlated to C-1 (δ_C_ 53.8) and C-9 (δ_C_ 37.0) by *g*HMBC experiments. Moreover, selective irradiation at δ_H_ 2.84 (H-1α) simplified the signals at δ_H_ 1.41 (H-2a), 1.13 (H-2b), and 1.93 (H-10), and evidenced a *cis* relationship between H-1 and H-10 (*J* = 2.5 Hz). Long-range coupling between H-6α (δ_H_ 2.52**)** and H-8α (δ_H_ 1.75) was also observed (*J* = 1.7 Hz) by the selective irradiation of these hydrogen frequencies. Based on all these data a 4,5-seco-guaiane structure can be suggested for **1**,* i.e.*, a bulnesol-type structure that was oxidized at C-4 and C-5. Spatial interactions between H-1 and H-2a, H-6α, H-7, H-9α, and H-10, as well as between H-8β and H-6β and 3H-14 observed by 1D-NOESY experiments allowed us to determine the conformation and the relative configuration as 1*S*,7*R*,10*S* (Figure 2). The high field chemical shift observed for H-8β (δ_H_ 1.10) is consistent with an endocyclic hydrogen protected by the carbonyl group at C-5. The spatial interactions between the methyl hydrogens 3H-14 and H-2a, H-8β, and H-9β confirmed this relative configuration.

**Scheme 1:**
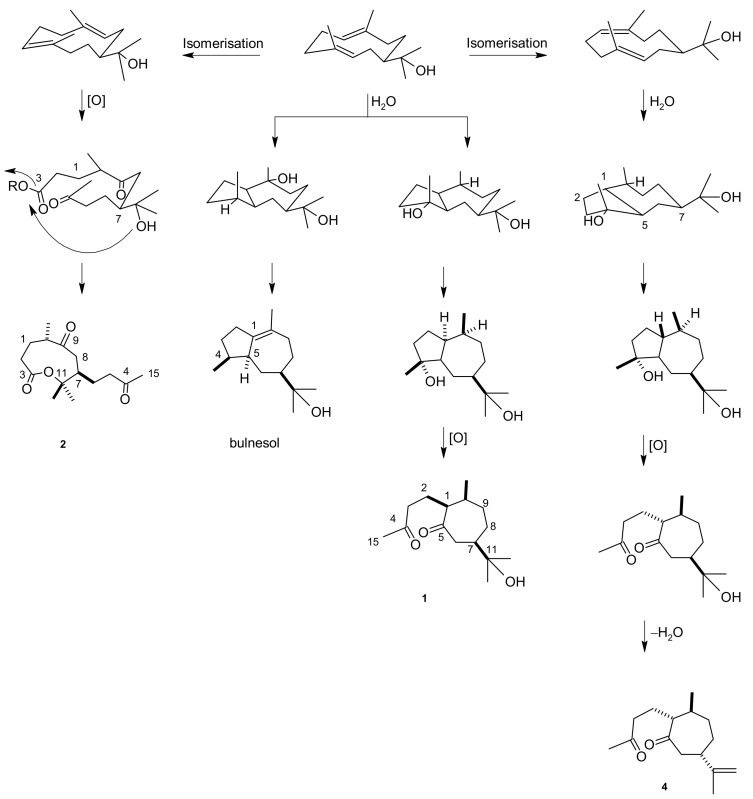
Proposed biogenetic pathway for sesquiterpenes **1**, **2**, and **4**.

A compound with this basic structure was previously obtained as a key intermediate in the synthesis of guaipyridine alkaloids from guaiol [12]. To date, however, except for the chemical shift of the methyl ketone hydrogens, no spectroscopic data for this compound have been reported in the literature. Chabrolidione A and its 1-*epi* isomer (**4**, Scheme 1), which have the same carbon skeleton as **1**, were isolated from the soft coral *Nephthea chabrolii *[13] and *Sinularia leptoclados *[14], respectively. However, they showed different relative configurations (at stereocenter C-10). Proposed biogenetic pathways for the formation of sesquiterpenes **1** and **4**, which may involve water addition and the cyclization of hedycaryol and its isomer followed by oxidation, are shown in Scheme 1. Sesquiterpene **1** was named 7-*epi*-11-hydroxychabrolidione A. 

**Figure 3:**
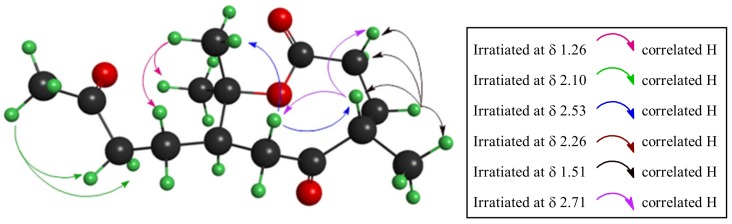
Selected nOe interactions for sesquiterpene **2**.

The ^13^C-NMR spectrum of **2** showed signals for 15 carbons, including two carbonyl groups at δ 215.4 and 208.0, and an acyl group at δ 171.0. The IR spectrum showed a broad and intense absorption band of these groups at ~ 1732 and 1707 cm^−1^, and did not show a characteristic OH absorption band. The HRMS spectrum of compound **2** showed *quasi*-molecular ions [M+Na]^+^ at *m/z* 291.1567 (calc for C_15_H_24_O_4_+Na 291.1567) and *m/z*. 269.1745 [M+H]^+^ (calc for C_15_H_25_O_4_ 269.1747). From these data, a total of four hydrogen deficiencies were determined for the structure, one of which was a ring, since no additional sp^2^ carbon was observed in the ^13^C-NMR spectrum. In addition, a carbinolic carbon (δ_C_ 85.0), five methylenes (δ_C_ 23.8, δ_H_ 1.57; δ_C_ 30.9, δ_H_ 1.51; δ_C_ 34.6, δ_H_ ~2.20; δ_C_ 42.2, δ_H_ 2.51; δ_C_ 43.0, δ_H_ 2.53 and 1.96), two methine groups (δ_C_ 47.7, δ_H_ 2.71; δ_C_ 49.2, δ_H_ 2.26), and four methyl groups (δ_C_ 18.8, 20.3, 25.7, 30.2 and δ_H_ 0.97, 1.26, 1.57, 2.10, respectively) were observed by an analysis of the ^1^H and ^13^C-NMR spectra, DEPT 135°, *g*HSQC, and *g*HMQC experiments (Table 2). A detailed analysis of ^1^H–^1^H COSY, selective proton decoupling (HOMODEC), and *g*HMBC experiments allowed us to determine the chemical shifts of the remaining hydrogens (H-1α, H-2β, H-6b) (Table 2). The *g*HMBC spectrum showed correlations between the carbonyl carbon at δ_C_ 208.0 (C-4) and the methyl hydrogens at δ_H_ 2.10 (3H-15) and the methylene hydrogens at δ_H_ 2.51 (2H-5). These latter were also correlated to the methine carbon at δ_C_ 49.2 (C-7). The carbinolic carbon and C-7 correlated with methyl hydrogens at δ_H_ 1.57 (3H-12) and 1.26 (3H-13) as well as with H-8α (δ_H_ 1.96). Selective irradiation at δ_H_ 2.26 (H-7) led to the simplification of the multiplets at δ_H_ 2.53 (H-8β), 1.96 (H-8α), 1.57 (H-6a), and 1.23 (H-6b). ^1^H–^1^H COSY experiment showed couplings between 2H-5 (δ_H_ 2.51) and H-6a and H-6b, as well as between H-10 (δ_H_ 2.71) and 3H-14 (δ_H_ 0.97), H-1α (δ_H_ 2.06), and H-1β (δ_H_ 1.51). 1D-TOCSY experiments evidenced long-distance coupling between H-10 and 2H-2 (δ_H_ ~2.2). The *g*HMBC spectra also showed correlations between the carbonyl carbon at δ 215.4 (C-9) and H-8α and 3H-14, which, in turn, were correlated to C-1 and C-10. The chemical shifts of the carbinolic carbon and the acyl group suggested the presence of a lactone. Based on all of these data, and with the aid of 1D-TOCSY experiments, a nine-membered lactone structure, with a 3-oxobutyl substituent, was established for **2**. Moreover, the coupling constant values (*J*) for the hydrogens that showed virtual couplings (H-1α, H-1β, H-2α, H-2β, Table 2) were determined by using the ACD/C+H NMR Predictors program (Advanced Chemistry Development, [15]) and confirmed by HOMODEC experiments. In addition, *g*NOESY experiments were used to determine the conformation of this sesquiterpene lactone based on spatial interaction between the hydrogens, as shown in Figure 3. In this conformation, the spatial interactions between H-8β and 3H-13 and H-10β could be justified. Besides, the anisotropic effects of the acyl group C-3 on the hydrogens CH_3_-12 and of carbonyl group C-9 on H-7 and H-lα, which are in *pseudo*-axial positions, could explain the high chemical shifts observed for these hydrogens. Therefore, the relative configuration for **2** was established as 7*R*,10*S*. Hence, sesquiterpene **2** has a new carbon skeleton and was named holostylactone. Its biosynthesis may involve oxidation at C-3,4, and C-9 of the key precursor hedycaryol, which leads to the breakage of the C-3,4 bound, followed by lactonization involving OH-11 and C-3 (Scheme 1).

**Figure 4:**
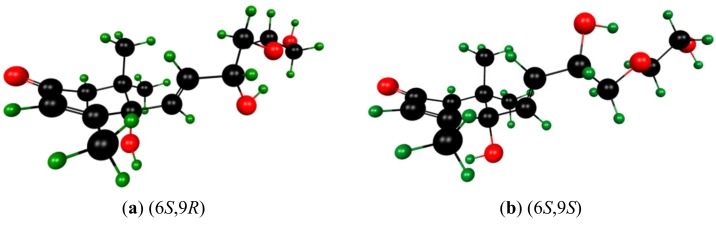
Conformations for compound **3**, (**a**) or (**b**).

HRMS spectrum of compound **3** showed a *quasi*-molecular ion [M+Na]^+^ at *m/z* 307.1525 (C_15_H_24_O_5_+Na). The ^1^H and ^13^C-NMR spectra of **3** (Table 3) showed signals for two methyl groups linked to a sp^3^ quaternary carbon (δ_H_ 0.91, 0.93; δ_C_ 23.9, 23.0), a methyl group (δ_H_ 1.82; δ_C_ 19.0) linked at the β position of an α,β-unsaturated carbonyl group (δ_H_ 5.77; δ_C_ 197.4, 125.5, 164.0), and a *trans* olefin (δ_H_ 5.75, 5.69; δ_C_ 129.9, 131.8) linked to carbinolic carbons (δ_C_ 71.6, 79.1 δ_C_). These data, together with the results of *g*COSY, *g*HMQC, *g*HMBC, and* g*NOESY experiments, led to a megastigmane carbon skeleton for **3**. Indeed, the ^1^H and ^13^C-NMR data of **3** (Table 3) were very similar to those published for cucumegastigmane I and the aglycone of cucumegastigmane II [16]. The main differences between their ^13^C-NMR spectral data were due to the signals observed for an O-CH_2_CH_2_OH group at C-10 of **3** (δ_C_ 66.1 and 60.2). The absolute configuration of cucumegastigmane I was determined by using a modified Mosher’s method and by application of the circular dichroism (CD) helicity rule on analogous compounds for determination of C-6 configuration [16]. Based on a comparison of the optical activities of **3** (CD Δε = +2.3 at 243 nm), cucumegastigmane I (CD Δε = +15.8 at 241 nm), and blumenol A (CD Δε = +9.6 at 243 nm) [17], the same absolute configuration could be inferred for C-6 of **3**,* i.e.*, (6*S*), since they showed positive Cotton effect at λ ~ 242 nm and follow the same helicity rule for π-π* enone Cotton effect [17]. Although the absolute configuration of C-9 remain to be determined, the structure established for **3** is (6*S*,7*E*)-6,9-dihydroxy-10-(2'-hydroxyethoxy)-4,7-megastigmadien-3-one (named holostymegastigmadienone) (Figure 4).

In addition to the NOESY experiments quantum chemical calculations were carried out at the B3LYP/STO-3G** level as fully described in the supplementary data. In order to have a reliable starting point for the calculations molecular structures with 7- and 9-membered rings were searched in the Cambridge Structural Database [18], basically two different conformations for these rings were found (Figure S8). Unrestricted optimizations have been carried out for these conformers. 

The four stationary structures found for the 7-membered ring have similar energies (Table S1 and Figure S9). Nevertheless, the NBO steric analysis (Table 4) indicates that only the **74**-conformer holds all spatial interactions noted by 1D-NOESY experiments. In fact the calculated **74**-conformer was used to draw Figure 2. For sesquiterpene **2 **also four conformers were found but in this case one of them, conformer **91**, is much more stable as shown in Table S1. Moreover, the NBO steric analysis shows that this conformer has the strongest spatial interactions for the ones displayed by the 1D-NOESY experiments (Table 4). This conformation was the one used for drawing Figure 3. 

From the foregoing it is clear that the molecular modeling studies support the 1D-NOESY experimental results, thus proving that the **74**- and **91**-conformer describe the 3D structures of the 7- and 9- membered ring compounds, respectively.

## 3. Experimental 

### 3.1. General Experimental Procedures

One-dimensional (^1^H, ^13^C, DEPT, HOMODEC, TOCSY, and *g*NOESY) and two-dimensional (^1^H–^1^H *g*COSY, *g*NOESY, *g*HMQC, *g*HSQC, and *g*HMBC) NMR experiments were performed on a Varian INOVA 500 spectrometer (11.7 T) at 500 MHz (^1^H), and 126 MHz (^13^C), using deuterated solvents (CDCl_3_ and DMSO-*d*_6_) (P 99.9% D) as an internal standards for ^13^C-NMR chemical shifts and residual solvent as an internal standard for ^1^H-NMR. δ values are reported relative to TMS. Mass spectra (ESI-MS) were obtained on a LCQ *Fleet–*Thermo Scientific, and flow injection into the electrospray source was used for LC-ESI-MS. High-resolution mass spectra (HRMS) were obtained on a Bruker Daltonics ultrOTOFq (ESI-TOFMS). IR spectra were obtained on a Perkin–Elmer 1600 FT-IR spectrometer using KBr discs. Optical rotations were measured on a Perkin Elmer 341-LC polarimeter. Ultraviolet (UV) absorptions were measured on a Perkin Elmer UV-vis Lambda 14P diode array spectrophotometer. HPLC analyses were performed using a Shimadzu liquid chromatograph (SPD-10 Avp), equipped with UV–vis and 341-LC polarimeter detectors, and using a Jasco LC-NetII/ADC, equipped with photodiode array (MD-2018 Plus) and CD (2095 Plus) detectors. The columns were RP-18 (Varian, C18, with a particle size of 5 *µ*m, 250 × 4.6 mm for analytical analysis, and 250 × 20 mm for semi-preparative analysis), and chromatograms were acquired at 336 and 254 nm. TLC: silica gel 60 PF_254_. 

### 3.2. Plant Materials

The plant materials were collected in Ituiutaba, MG, Brazil, in February 2008, and identified as *Holostylis reniformis *Duch. by Dr. Vinícius C. Souza and Dr. Lindolpho Cappellari Jr. A voucher specimen (ESA 88282/2008) was deposited at the herbarium of the Escola Superior de Agricultura, Luiz de Queiroz (ESALQ), Piracicaba, SP, Brazil. The material was separated according to the plant parts and dried (~45 °C).

### 3.3. Extraction and Isolation of the Chemical Constituents

The roots (107.9 g) were ground and exhaustively extracted successively at room temperature with hexanes, acetone, and ethanol [4 × (~200 mL, 2 days) each solvent]. The residues were extracted with ethanol in a Soxhlet apparatus and the extracts were individually concentrated. The crude hexane extract (6.17 g) was subjected to CC (6.0 by 40.0 cm, silica gel 60H, 127.3 g, hexanes/EtOAc gradient, 19:1 to 100% EtOAc) to give 28 fractions (*ca. *125 mL each) as previously described [3]. Fraction 5 (50.0 mg) gave bulnesol. Fraction 20 (280.0 mg) gave **1** (19.3 mg) after semi-preparative HPLC (MeOH–H_2_O, 3:2).

The acetone extract from roots (3.73 g) was also subjected to CC to give 26 fractions, as previously described [4]. Fraction 17 (15.5 mg) was partially dissolved with CH_3_CN and the soluble-portion was subjected to HPLC (MeOH-H_2_O 7:3) to give **3** (11.6 mg). Fraction 23 (9.0 mg) was washed with MeOH and the resulting soluble-portion gave sitosterol-3-*O*-β-D-glucopyranoside (3.0 mg).

*rel.-(2S,3S,6R)-6-(2-Hydroxypropan-2-yl)-3-methyl-2-(3-oxobutyl)cycloheptanone* (7-*epi*-11-*hydroxy-chabrolidione A*, **1**): Colorless oil; [α${]}_{D}^{25}$ +50.7 (*c* 0.9, CHCl_3_); IR (KBr) ν_max_ 3440, 2972, 2925, 2875, 1707, 1376 cm^−1^; ^1^H and ^13^C-NMR (CDCl_3_) see Table 1; HRESIMS (probe) 4.5 eV, positive mode, *m/z* (rel. int.): 277.1774 [M+Na]^+^ (C_15_H_26_O_3_+Na) (94) (calculated for C_15_H_26_O_3_+Na = 277.1774), 237.18 [M+H−H_2_O]^+^ (100); ESIMS 20 eV, positive mode, *m/z* (rel. int.): 237 (100), 235 (35).

*rel.-(5S,8R)-5,9,9-Trimethyl-8-(3-oxobutyl)oxonane-2,6-dione* (holostylactone, **2**): Colorless oil; [α${]}_{D}^{25}$ −20.0 (*c* 1.0, CHCl_3_); IR (KBr) ν_max_ 2912, 2862, 1732, 1707, 1389, 1100 cm^−1^; ^1^H and ^13^C-NMR (CDCl_3_) see Table 2; HRESIMS (probe) 4.5 eV, positive mode, *m/z* (rel. int.): 291.1567 (calc for C_15_H_24_O_4_+Na = 291.1567) (100) and *m/z* 269.1745 [M+H]^+^ (calc for C_15_H_25_O_4_ 269.1747) (60); ESIMS 20 eV, positive mode, *m/z* (rel. int.): 269 (100), 251 (55), 233, [M+Na−H_3_CCOCH_3_] (18).

*(6S,7E,9S)-6,9-Dihydroxy-10-(2'-hydroxyethoxy)-4,7-megastigmadien-3-one *(holostymegastigmadienone, **3**): Yellow oil; [α${]}_{D}^{25}$ +68.6 (*c* 0.9, MeOH); IR (KBr) ν_max_ 3452, 1666, 1643, 1384 cm^−1^; ^1^H and ^13^C-NMR (DMSO-*d*_6_) see Table 3; HRESIMS (probe) 4.5 eV, positive mode, *m/z* (rel. int.): 307.1525 [M+Na]^+^ (100) (calculated for C_15_H_24_O_5_+Na = 307.1522); ESIMS 20 eV, positive mode, *m/z* (rel. int.): 307 (38), 277 (87), 275 (100), 237 (43), 205 (47), 130 (24). CD (MeOH, *c *0.1): [θ]_221_ 0, [θ]_243_ + 7590, [θ]_271 _0, [θ]_312_−1815.

## 4. Conclusions 

*H. reniformis* can synthesize a variety of seco compounds, including lignans and sesquiterpenes. Among them, bulnesol and two unusual 4,5-seco-guainanes, which may be biogenetic derivatives of hedycaryol, were isolated from the roots of this species. In addition, a new megastigmadienone was isolated.

## Figures and Tables

**Table 1:** NMR Spectroscopic Data for Compound **1** (CDCl_3_, 11.7 T).

Position	δ_C_, type ^a^	δ_H_ (*J* in Hz) ^b^	*g*HMBC ^c^
1α	53.8, CH	2.84, ddd (9.5, 4.5, 2.5)	14
2a	24.2, CH_2_	1.41, dddd (14.5, 8.5, 6.0, 4.5)	
2b		1.13, m	
3a	41.7, CH_2_	2.37, ddd (17.0, 8.5, 6.0)	
3b		2.19, ddd (17.0, 9.0, 8.5)	
4	208.8, C		3a, 3b, 15
5	213.4, C		6β
6 α	47.0, CH_2_	2.52, ddd (17.5, 4.5, 1.7)	
6β		2.13, dd (17.5, 12.0)	
7	46.4, CH	1.84, dddd (12.0, 10.0, 4.5, 1.5)	12, 13
8α	25.1, CH_2_	1.75, m	
8β		1.10, m	
9α	37.0, CH_2_	1.73, m	8β, 14
9β		2.04, m	
10	34.9, CH	1.93, m	14, 2b and/or 8β
11	73.0, C		12, 13 and/or 8β
12	26.6, CH_3_	1.13, s	13
13	26.5, CH_3_	1.10, s	12
14	14.0, CH_3_	0.70, d (7.5)	
15	29.8, CH_3_	2.04, s	

^a^ Chemical shifts and multiplicities were determined with the assistance of DEPT and *g*HMQC experiments; ^b^ Multiplicities were determined with the assistance of ^1^H-^1^H COSY, ^1^H-^1^H TOCSY and HOMODEC experiments; ^c^ *g*HMBC correlations, optimized for 8 Hz, are from carbon stated to the indicated hydrogen(s).

**Table 2:** NMR Spectroscopic Data for compound **2** (CDCl_3_, 11.7 T).

H/C	δ_C_, type ^a^	δ_H_ (*J* in Hz) ^b, c^	*g*HMBC ^d^
1α	30.9, CH_2_	2.06, dddd ^c^ (14.5, 9.8, 9.3, 6.5)	14
1β		1.51, dddd ^c^ (14.5, 8.5, 2.5, 1.5)	
2α	34.6, CH_2_	2.22, ddd ^c^ (15.0, 6.5, 1.5)	
2β		2.19, ddd ^c^ (15.0, 9.8, 8.5)	
3	171.0, C		
4	208.0, C		5a, 5b, 15
5a, 5b	42.2, CH_2_	2.51 br, t (7.5)	15
6a	23.8, CH_2_	1.57, ddt (14.0, 2.0, 7.5)	8β
6b		1.23, ddt (14.0, 11.5, 7.0)	
7	49.2, CH	2.26, ddt (11.5, 11.0, 2.0)	5a, 5b, 8α, 12, 13
8α	43.0, CH_2_	1.96, dd (12.5, 2.0)	
8β		2.53, dd (12.5, 11.0)	
9	215.4, C		8α, 14
10	47.7, CH	2.71, ddq (9.3, 2.5, 7.0)	14
11	85.0, C		8α, 13, 12 and/or 6a
12	25.7, CH_3_	1.57, s	13
13	20.3, CH_3_	1.26, s	12
14	18.8, CH_3_	0.97, d (7.0)	
15	30.2, CH_3_	2.10, s	

^a^ Chemical shifts and multiplicities were determined with the assistance of DEPT and *g*HMQC experiments; ^b^ Multiplicities were determined with the assistance of ^1^H–^1^H COSY, ^1^H–^1^H TOCSY and HOMODEC experiments; ^c^ Calculated values; ^d^ *g*HMBC correlations, optimized for 8 Hz, are from carbon stated to the indicated hydrogen(s).

**Table 3:** NMR Spectroscopic Data for Compound **3** (DMSO-*d*_6_, 11.7 T).

H/C	δ_C_, type ^a^	δ_H_ (*J* in Hz) ^b^	*g*HMBC ^c^
1	40.9, C		11, 12
2	49.4, CH_2_	2.05, d (16.0)	11, 12
2		2.39, d (16.0)	
3	197.4, C		
4	125.5, CH	5.77, q (2.5)	13
5	164.0, C		13
6	79.1, C		11, 12, 13, 4, 7, OH
7	129.9, CH	5.75, d (17.0)	
8	131.8, CH	5.69, dd (17.0, 5.0)	
9	71.6, CH	4.00, m	7
10	72.2, CH_2_	3.30–3.40, m	1'
11	23.0, CH_3_	0.93, s	12
12	23.9, CH_3_	0.91, s	11
13	19.0, CH_3_	1.82, d (1.5)	4
1'	66.1, CH_2_	3.30–3.40, m	
2'	60.2, CH_2_	3.30–3.40, m	1'

^a^ Chemical shifts and multiplicities were determined with the assistance of DEPT and *g*HMQC experiments; ^b^ Multiplicities were determined with the assistance of ^1^H–^1^H COSY experiments; ^c^ *g*HMBC correlations, optimized for 8 Hz, are from carbon stated to the indicated hydrogen(s).

**Table 4:** NBO Steric Exchange Energies (kcal.mol^−1^)—Conformation.

**7-membered ring**					
Spatial Interaction	71	72	73	74
····–C1–H1…. H2a–C2–·····	0.79	1.52	1.17	0.68
····–C1–H1… H6α–C6–·····	1.00	0.09	----	0.19
····–C1–H1…. H7–C7–·····	----	----	0.04	0.59
····–C1–H1…. H9α–C9–·····	0.65	----	0.67	0.43
····–C1–H1..... H10–C10–····	1.81	1.74	2.07	0.99
····–C2–H2b… CH_3_(14)–····	0.99	----	0.89	0.72
····–C8–H8β… H6β–C6–····	----	----	0.63	0.27
····–C8–H8β.... CH_3_(14)–····	1.39	----	0.78	0.54
····–C8–H8β… O=C5–····	----	0.65	----	0.15
····–C8–H8β….O-H····	2.10	----	----	0.12
····–C9–H9β... .CH_3_(14)–····	0.30	0.76	0.40	0.75
**9-membered ring**					
	91	92	93	94
····–C1–H1α… O=C9–·····	1.21	----	0.02	0.01
···–C7–H7…. O=C9–·····	0.59	----	----	0.03
····–C8–H8β….H10–C10–·····	0.97	0.02	----	0.57
····–C8–H8β….CH_3_(13)– ·····	0.62	0.17	0.55	1.91
····–CH_3_(12)….O=C4–·····	11.04	9.24	----	8.18
····–CH_3_(12)….O=C3–O–·····	1.49	1.32	0.59	0.93

## Supplementary Materials

Supplementary materials can be accessed at: http://www.mdpi.com/1420-3049/17/12/14046/s1.
