# Supplementary material for: 4,5-Seco-Guaiane and a Nine-Membered Sesquiterpene Lactone from Holostylis reniformis
Source: Molecules. 2012 Nov 27;17(12):14046–57. doi: 10.3390/molecules171214046 (PMC6268306; doi:10.3390/molecules171214046)
Supplement: Supplementary file 1 [file molecules-17-14046-s001.pdf]

Supplementary Materials

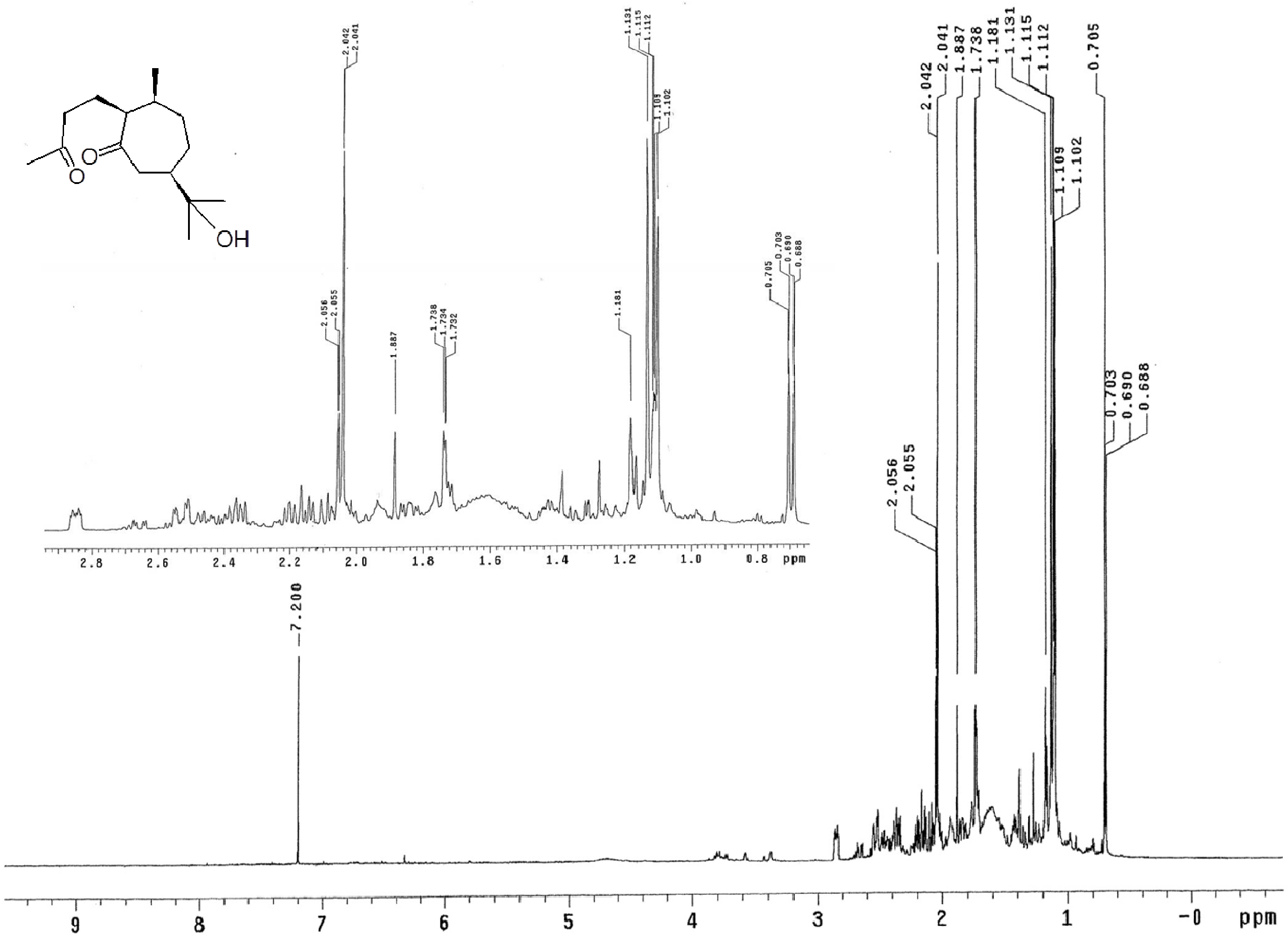

Figure S1. ^{1}H-NMR (500 MHz, $CDCl_3$) spectrum of the new compound **1**.

Figure S2. ^{13}C-NMR (126 MHz, $CDCl_3$) spectrum of the new compound **1**.

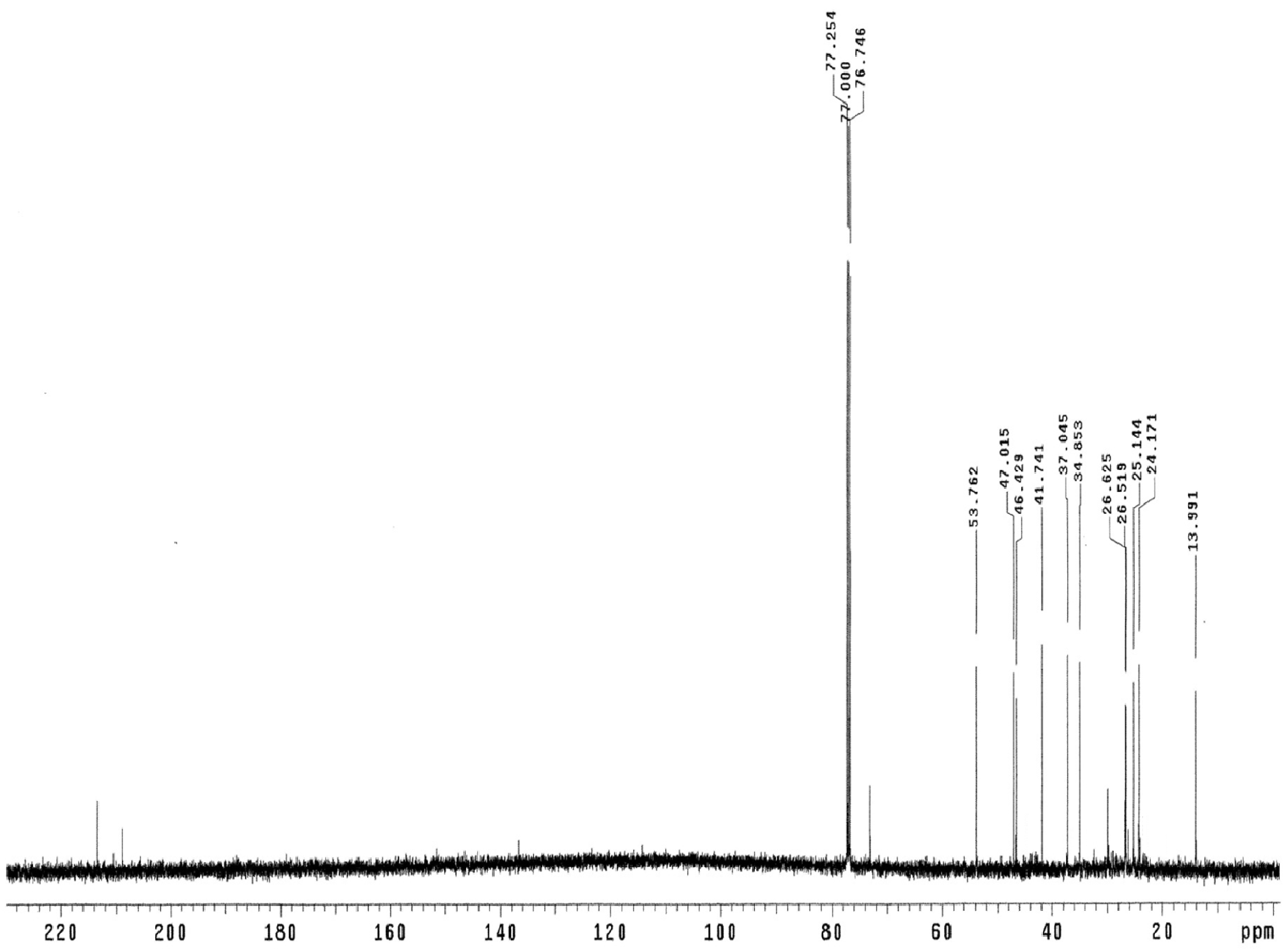

Figure S3. ^{1}H-NMR (500 MHz, $CDCl_3$) spectrum of the new compound **2**.

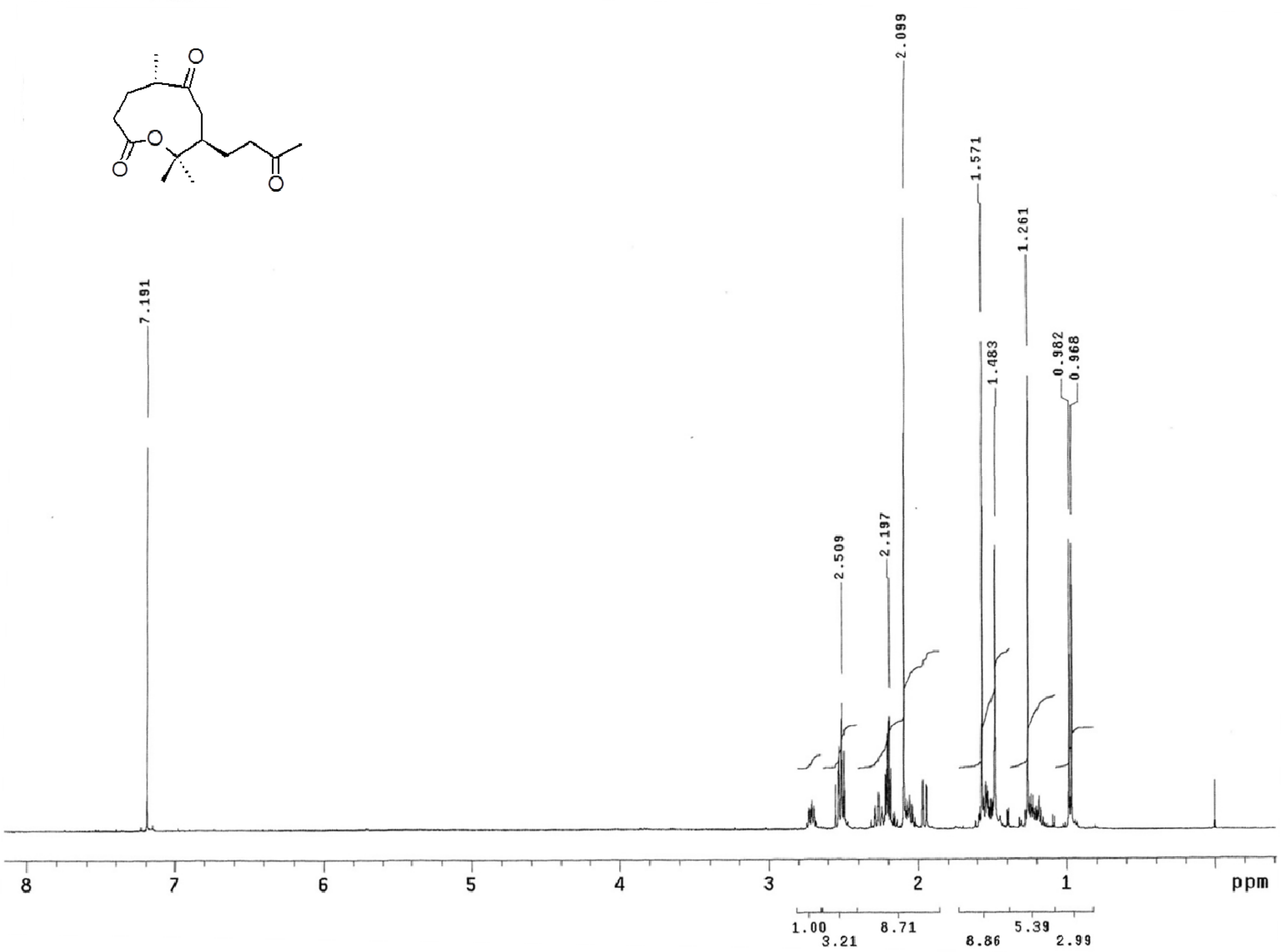

Figure S4. Amplified ^{1}H-NMR (500 MHz, $CDCl_3$) spectrum of the new compound **2**.

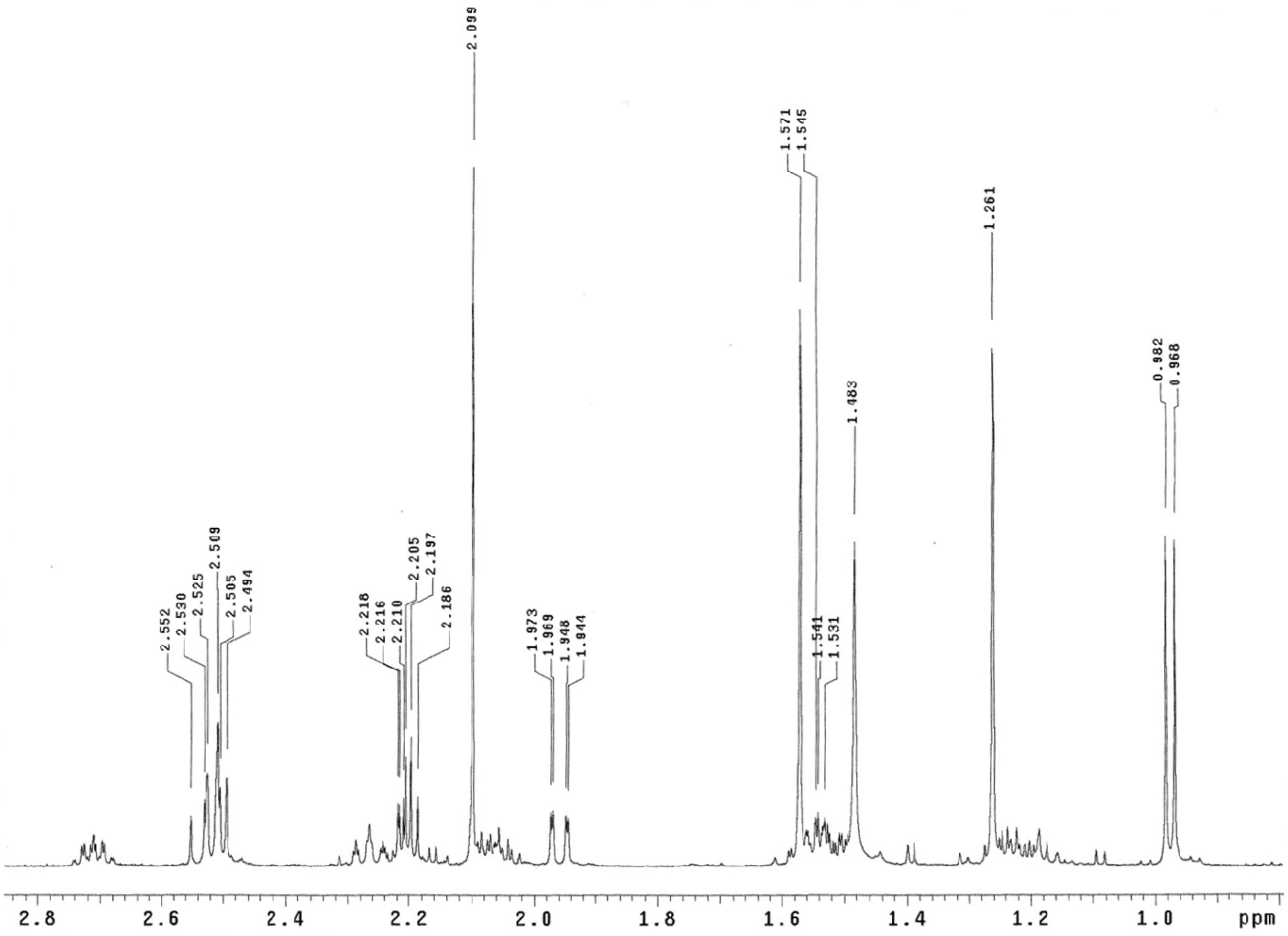

Figure S5. ^{13}C-NMR (126 MHz, $CDCl_3$) spectrum of the new compound **2**.

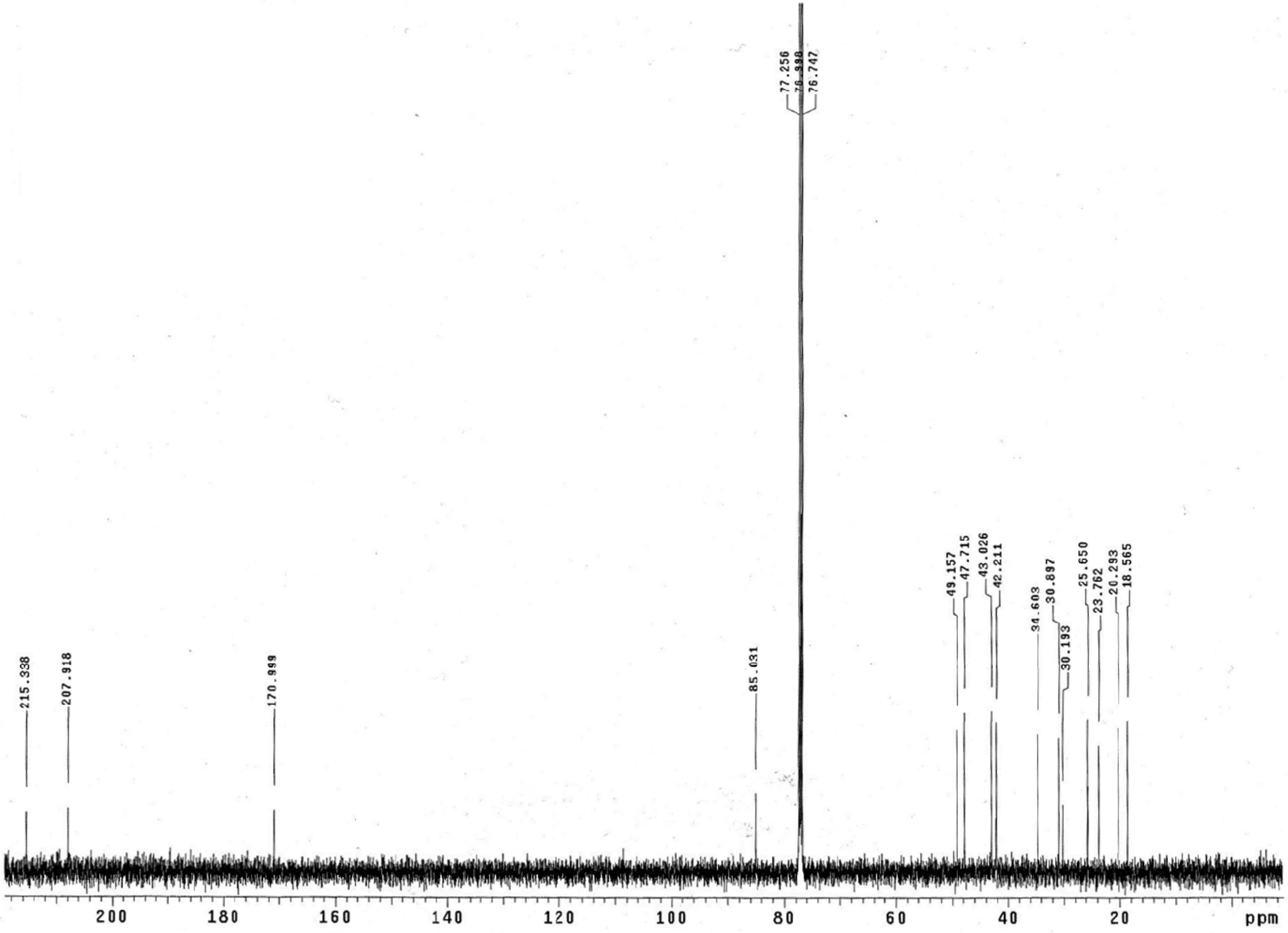

Figure S6. ^{1}H-NMR (500 MHz, DMSO- d_6) spectrum of the new compound **3**.

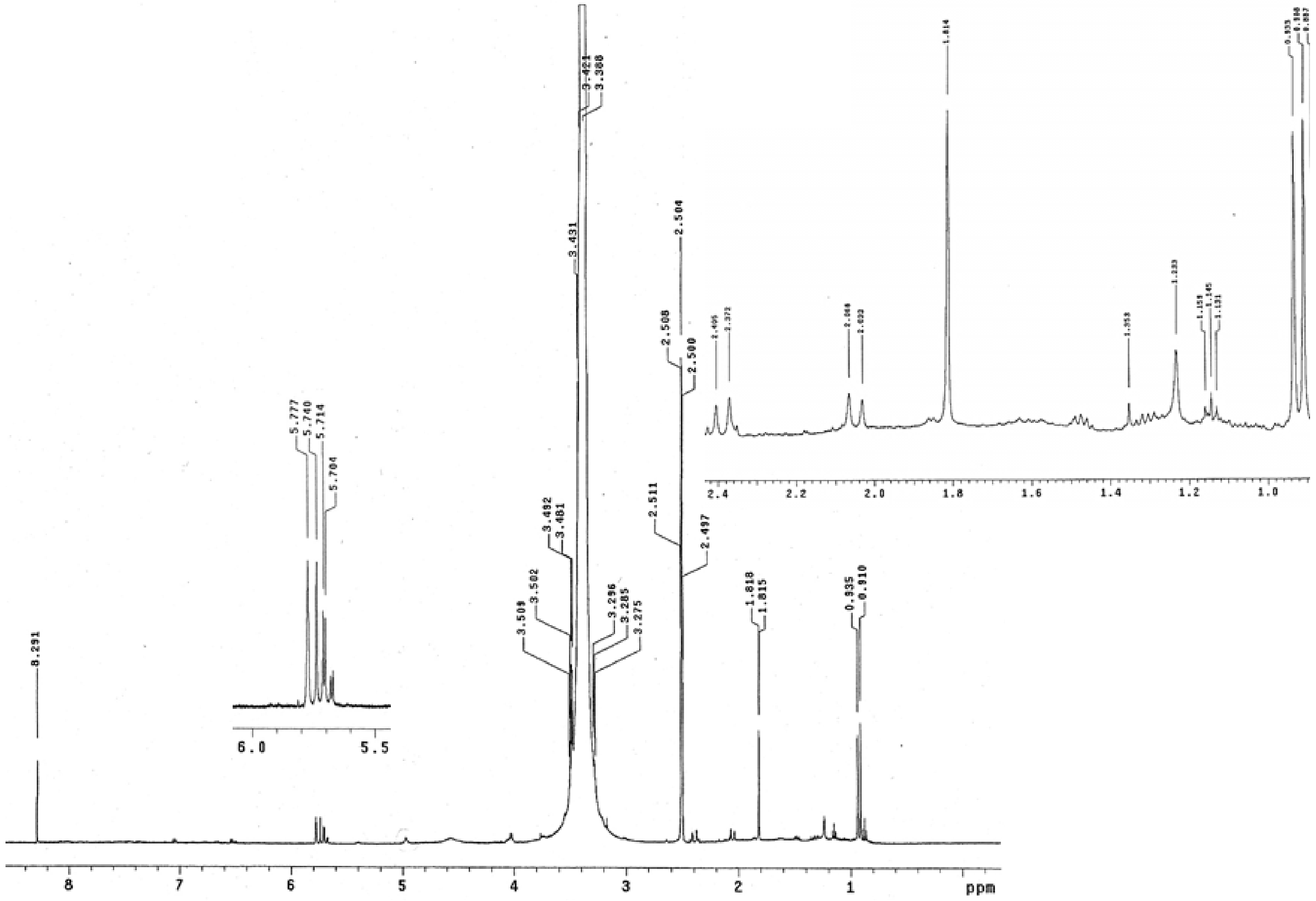

Figure S7. ^{13}C-NMR (126 MHz, DMSO-d_6) spectrum of the new compound **3**.

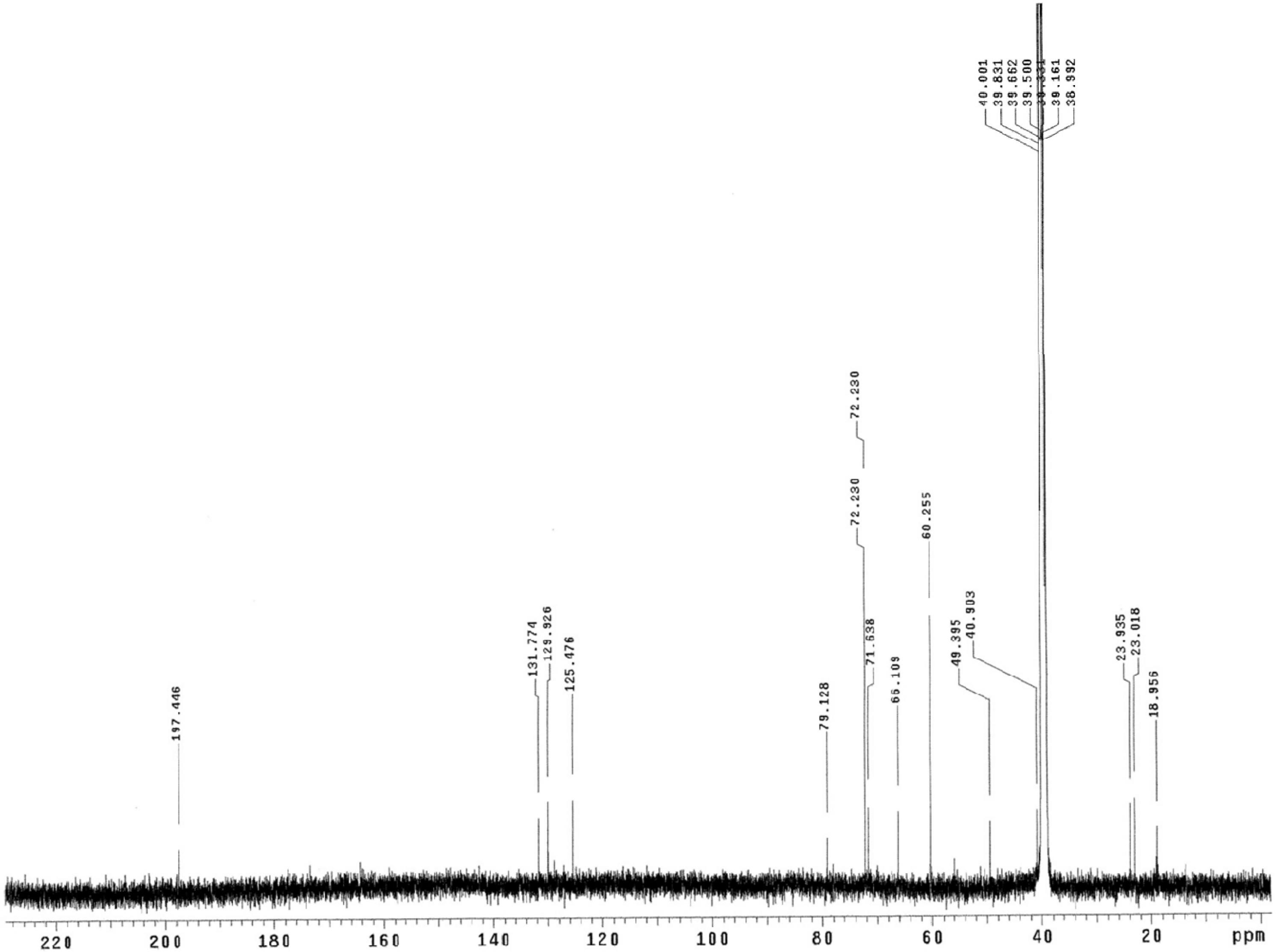

Computational Details

Calculations have been carried out using PC GAMESS package at the B3LYP/STO-3G** level and wxMacMolPlt software for structure visualization [1–6]. Optimization algorithm was based on the Quadratic Approximation (QA) and the threshold gradient value was 10^{-5} a.u. [7]. Vibrational analysis showed all real frequencies asserting the stationary points as minima [7]. The NBO donor-acceptor pairs were checked and steric energies were calculated [8–12].

Figure S8. Conformations found in the Cambridge Structural Database.

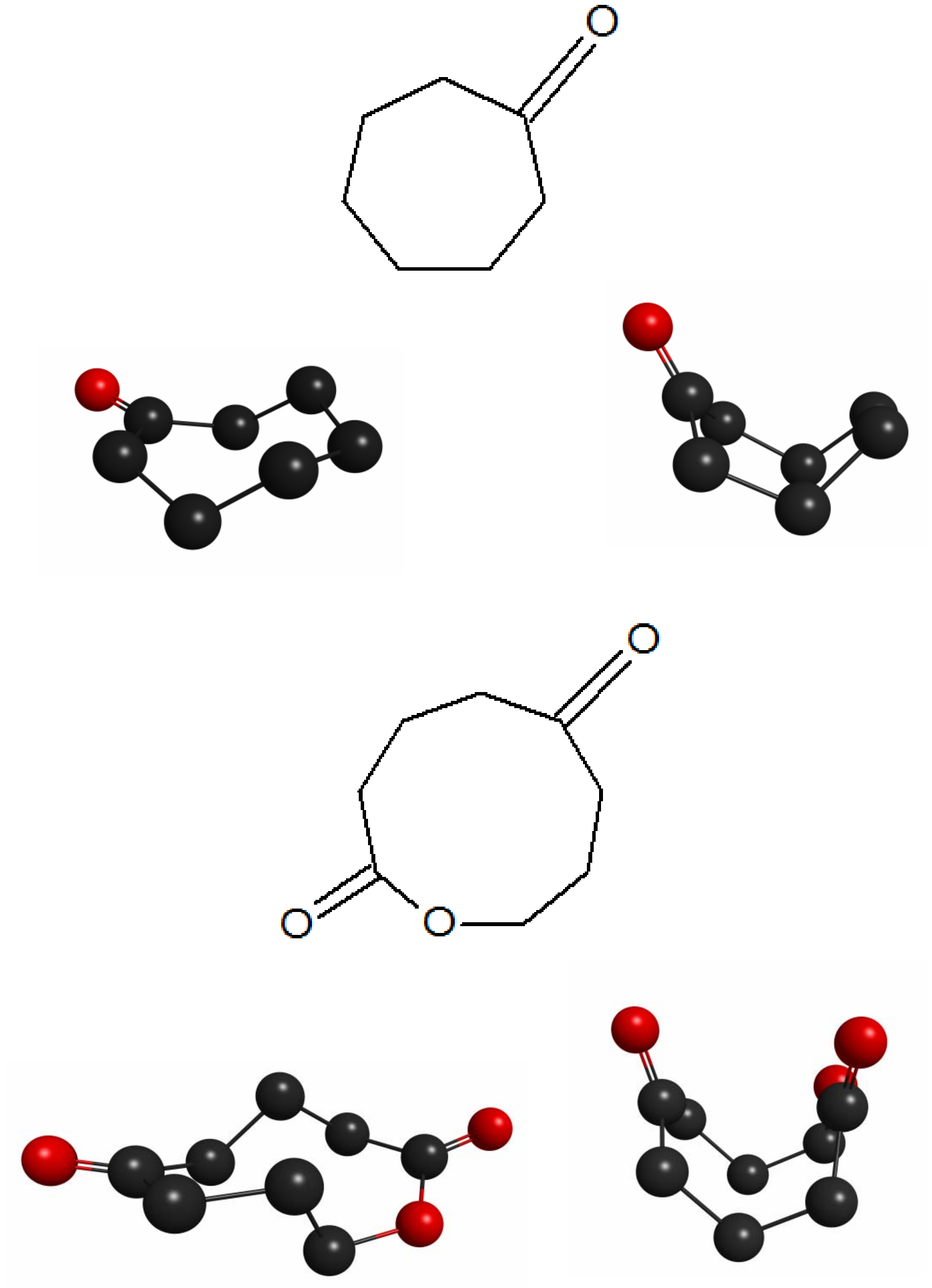

Table S1. Relative Energies ($kcal.mol^{-1}$)—Conformation.

**7-membered ring**			
Conformer	ΔG° (298.15 K)	ΔE_{ZPE}	ΔE
**71**	0.0	0.0	0.0
**72**	0.0	0.2	0.7
**73**	0.4	1.2	2.0
**74**	−0.6	1.1	2.4
**9-membered ring**			
Conformer	ΔG° (298.15 K)	ΔE_{ZPE}	ΔE
**91**	0.0	0.0	0.0
**92**	4.3	4.0	3.7
**93**	14.4	15.3	15.9
**94**	22.6	22.3	22.0

Figure S9. Optimized structures for the 7-membered ring compound.

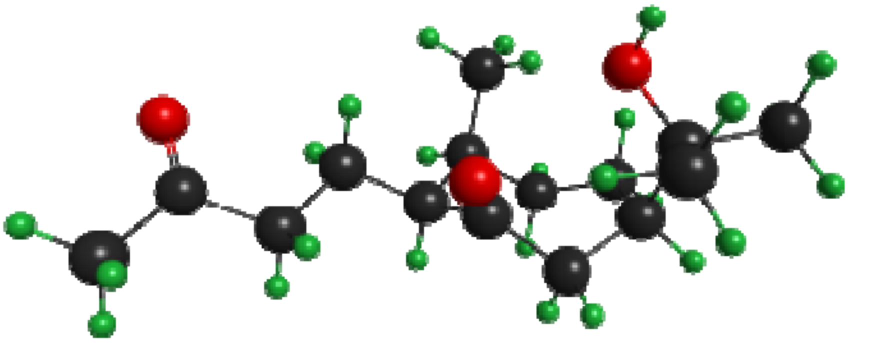

71

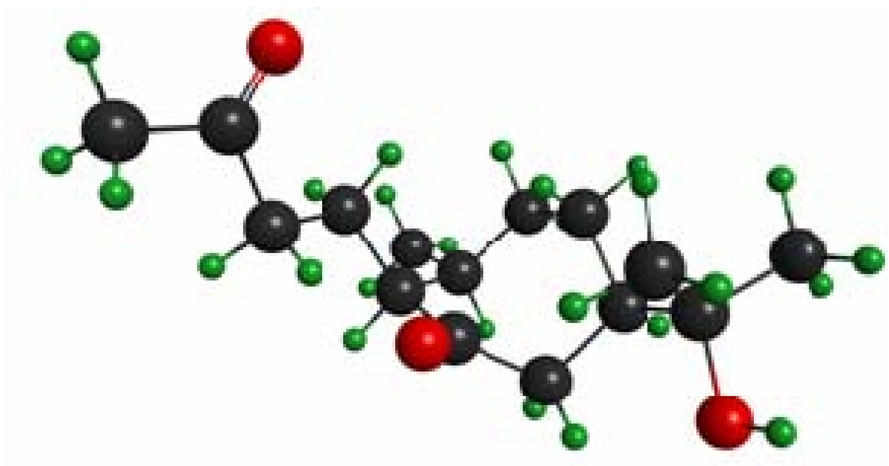

72

Figure S9. *Cont.*

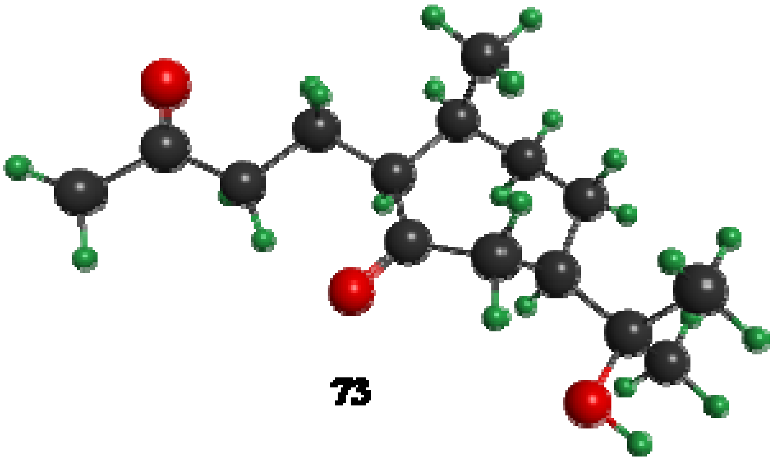

73

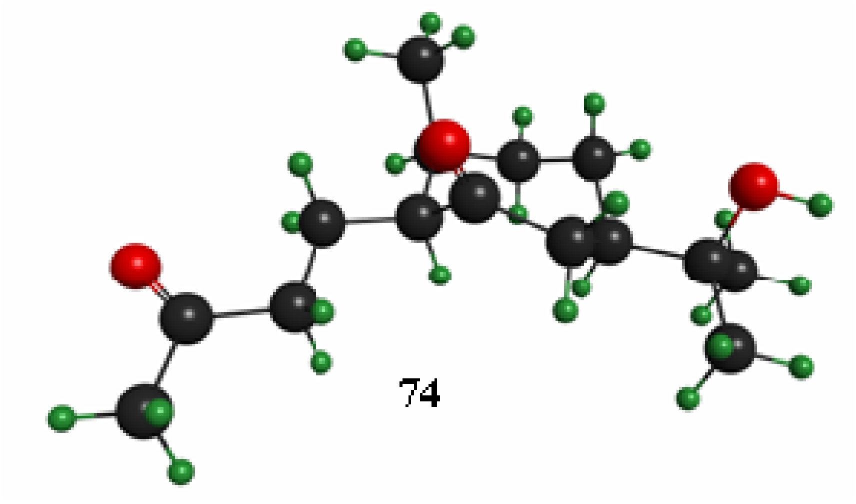

74

Figure S10. Optimized structures for the 9-membered ring compound.

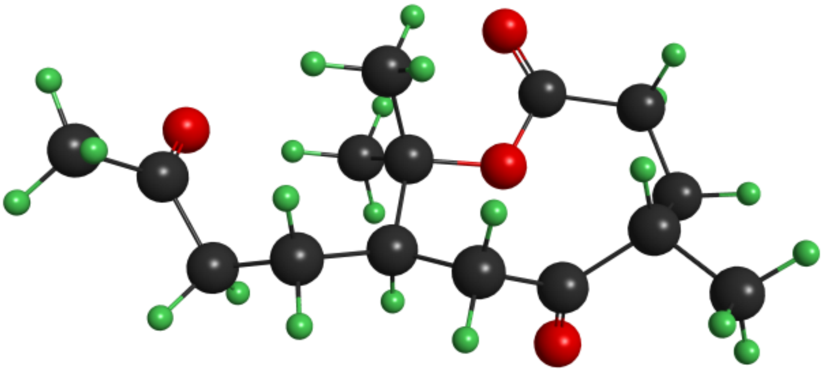

Figure S10. *Cont.*

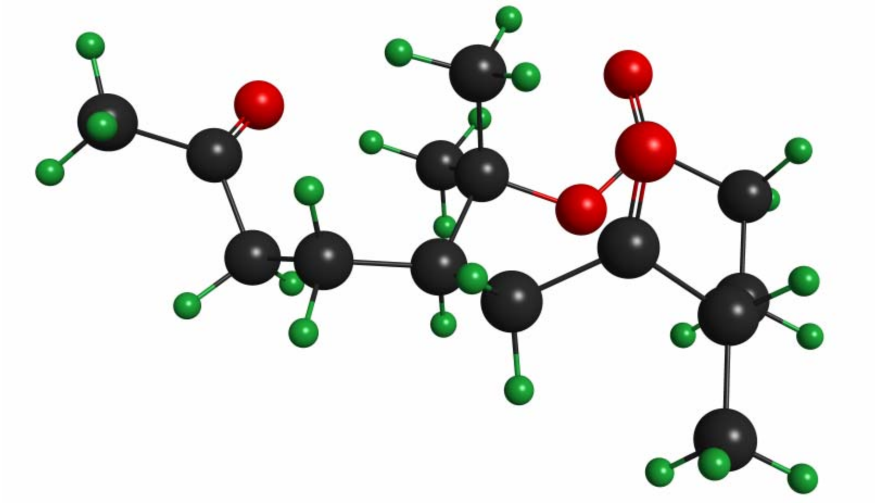

92

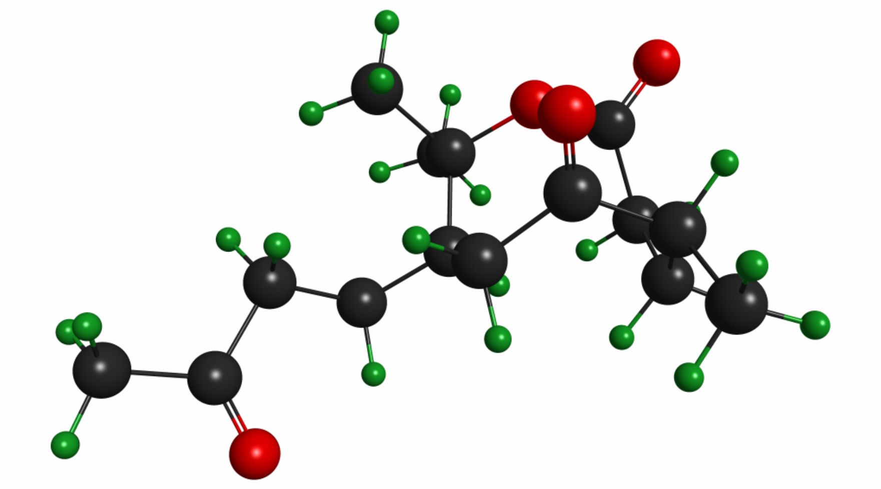

93

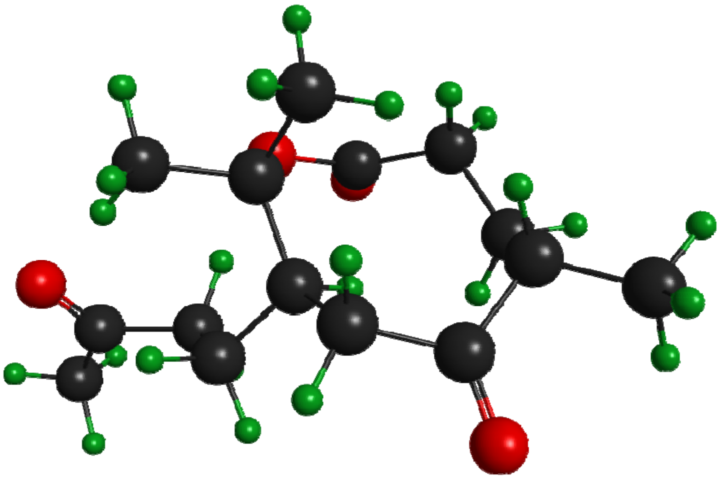

94

References

1. PC GAMESS. Available online: http://classic.chem.msu.su/gran/gamess/index.html (accessed on 31 November 2011).
2. Becke, A.D. Density-functional thermochemistry. III. The role of exact exchange. *J. Chem. Phys.* **1993**, *98*, 5648–5652.
3. Hehre, W.J.; Stewart, R.F.; Pople, J.A. Self-consistent molecular-orbital methods. I. Use of gaussian expansions of slater-type atomic orbitals. *J. Chem. Phys*. **1969**, *51*, 2657–2664.
4. Hehre, W.J.; Ditchfield, R.; Stewart, R.F.; Pople, J.A. Self-consistent molecular orbital methods. IV. Use of gaussian expansions of slater-type orbitals. Extension to second-row molecules. *J. Chem. Phys.* **1970**, *52*, 2769–2773.
5. Collins, J.B.; Schleyer, P. von. R.; Binkley, J.S.; Pople, J.A. Self-consistent molecular orbital methods. XVII. Geometries and binding energies of second-row molecules. A comparison of three basis sets. *J. Chem. Phys.* **1976**, *64*, 5142–5151.
6. Bode, B.M.; Gordon, M.S. Macmolplt: **A** graphical user interface for GAMESS. *J. Mol. Graphics Mod*. **1998**, *16*, 133–138.
7. Jensen, F. Locating transition structures by mode following: A comparison of six methods on the Ar_8 Lennard-Jones potential. *J. Chem. Phys*. **1995**, *102*, 6706–6718.
8. Foster, J.P.; Weinhold, F. Natural hybrid orbitals. *J. Am. Chem. Soc*. **1980**, *102*, 7211–7218.
9. Reed, A.E.; Weinstock, R.B.; Weinhold, F. Natural population analysis. *J. Chem. Phys.* **1985**, *83*, 735–746.
10. Reed, A.E.; Weinhold, F. Natural localized molecular orbitals. *J. Chem. Phys.* **1985**, *83*, 1736–1740.
11. Badenhoop, J.K.; Weinhold, F. Natural bond analysis of steric interactions. *J. Chem. Phys.* **1997**, *107*, 5406–5421.
12. Badenhoop, J.K.; Weinhold, F. Natural steric analysis of internal rotation barriers. *Int. J. Quantum Chem*. **1999**, *72*, 269–280.
